# Co-culture of ASCs/EPCs and dermal extracellular matrix hydrogel enhances the repair of full-thickness skin wound by promoting angiogenesis

**DOI:** 10.1186/s13287-021-02203-1

**Published:** 2021-02-12

**Authors:** Shuang Lin, Xiaoning He, Yuanjia He

**Affiliations:** 1 Department of Plastic Surgery, Shengjing Hospital of China Medical University, Shenyang, China; 2 Department of Stomatology, The Fourth Affiliated Hospital of China Medical University, Shenyang, Liaoning China

**Keywords:** ASCs, EPCs, ECM, Vascularization

## Abstract

**Background:**

The repair of large-scale full-thickness skin defects represents a challenging obstacle in skin tissue engineering. To address the most important problem in skin defect repair, namely insufficient blood supply, this study aimed to find a method that could promote the formation of vascularized skin tissue.

**Method:**

The phenotypes of ASCs and EPCs were identified respectively, and ASCs/EPCs were co-cultured in vitro to detect the expression of dermal and angiogenic genes. Furthermore, the co-culture system combined with dermal extracellular matrix hydrogel was used to repair the full-scale skin defects in rats.

**Result:**

The co-culture of ASCs/EPCs could increase skin- and angiogenesis-related gene expression in vitro. The results of in vivo animal experiments demonstrated that the ASCs/EPCs group could significantly accelerate the repair of skin defects by promoting the regeneration of vascularized skin.

**Conclusion:**

It is feasible to replace traditional single-seed cells with the ASC/EPC co-culture system for vascularized skin regeneration. This system could ultimately enable clinicians to better repair the full-thickness skin defects and avoid donor site morbidity.

## Introduction

The repair of large-scale full-thickness skin defects represents a challenging problem in skin tissue engineering [1, 2]. At present, skin tissue engineering technology has made great progress, but there are still some obstacles, such as obvious scarring of new skin, large amount of tissue shrinkage, slow skinization speed, and difficulty in constructing full-thickness skin tissue [3–5]. The main reason is that the implanted tissue-engineered skin lacks angiogenesis and is unable to obtain sufficient nutrition, which limits the repair of large-scale full-thickness skin defects. Previous studies have confirmed that angiogenesis is an important factor affecting the process of skin formation in the body, and capillaries are involved in the process of skin regeneration [6]. At the site of new skin formation, fibroblasts were found near vascular endothelial cells, suggesting that angiogenesis and skin regeneration are interdependent [7]. Meanwhile, in the process of skin formation, restricted angiogenesis will lead to poor skin healing, increased scar tissue, and delayed wound healing. In the repair of small-scale skin defects, skin tissue has a strong ability to regenerate. This regenerative ability is achieved by the vascular system around the defect area, which continuously recruits nearby fibroblasts and related bioactive factors for skin repair and provides nutritional support for the repair process [8]. However, in the case of large-scale skin defects, severe damage to the surrounding vascular system will affect the recruitment of nearby fibroblasts and the transportation of nutrients, resulting in the limitation of the regeneration process of skin tissues and failure to complete the repair of large-scale skin defects [9]. Therefore, in the process of repairing skin defects, it is essential to achieve vascularized skin regeneration.

The application of endothelial progenitor cells (EPCs) is a potential method to achieve vascularization [10]. EPCs are precursor cells of vascular endothelial cells and have the ability to proliferate, migrate, and differentiate into cells arranged along the lumen of the blood vessels [11]. Previous studies have found that EPCs participate in the process of skin formation and repair, and vascular endothelial cells enhance the activity of pre-fibroblasts and play an important role in the process of skin regeneration [12, 13]. Adipose-derived stem cells (ASCs) are extracted from adipose tissue and have high proliferation and growth characteristics and multi-differentiation potential [14]. Compared with other tissue-derived stem cells, ASCs have the unique advantages of extensive tissue sources, easy availability, less damage to the donor, and high safety. As a type of seed cell for skin tissue engineering, ASCs have been proven to promote skin tissue regeneration [15]. As confirmed in previous studies, ASCs can accelerate the recruitment of EPCs, enhance their angiogenesis capability, and promote the formation of new blood vessels. Besides, ASCs can secrete a variety of biologically active factors such as vascular endothelial growth factor (VEGF) and epidermal growth factor (EGF), which are beneficial to enhance the viability of endothelial cells to promote angiogenesis. Therefore, co-culture of ASCs/EPCs may promote the formation of vascularized skin through cell-cell interaction [16, 17].

To study the effect of co-culture of ASCs and EPCs on the construction of vascularized skin tissue, the phenotype of ASCs and EPCs was first identified, and then it was determined whether the co-culture of ASCs and EPCs could promote the differentiation of skin and vascular endothelial cells in vitro. Furthermore, the ASC/EPC co-culture system combined with dermal ECM gel material was used to repair severe skin defects in rats, and analyzed by gross, histology, and immunofluorescence assays. The results indicated that the co-culture of ASCs and EPCs could enhance skin regeneration and angiogenesis and significantly promote the repair of large-scale skin defects. It is feasible to use the ASC/EPC co-culture system to replace traditional single-seed cells for skin tissue engineering.

## Materials and method

### Preparation and culture of ASCs and EPCs

Animal procedures were conducted in accordance with the protocol approved by IACUC of China Medical University. Sprague-Dawley (SD) rats, 4 weeks old, were euthanized by CO_2_. The subcutaneous white adipose tissue of the inguinal region was harvested, washed with phosphate-buffered saline (PBS), and minced by sterile surgical scissors. The minced tissue was digested with 0.1% collagenase type I (Gibco) at 37 °C for 1 h and then centrifuged at 1000 rpm for 5 min. The upper layer of the undigested fat and fibrous connective tissue was removed, and the cell pellet in the lower layer was resuspended and filtered through a 70-μm filter. After another centrifugation for 5 min, the cell pellet was resuspended in the culture medium consisting of low-glucose Dulbecco’s modified Eagle medium (DMEM) (Hyclone), 10% fetal bovine serum (FBS) (Gibco), and 1% penicillin/streptomycin. The cell suspension was seeded into a 25-cm^2^ flask and cultured at 37 °C with 5% CO_2_ in a humidity atmosphere. The medium was changed 24 h after inoculation, and then replaced every 2–3 days, and the cells were passaged after 80% confluence. The ASCs at passage 3 were collected for the following experiments.

After sacrifice with CO_2_, the bone marrow in the femurs of 4-week-old SD rats was blown out and beaten evenly. Following the centrifugation with Histopaque-1083 (Sigma), the cell pellets were collected and resuspended in a mononuclear cell separation solution. Then, CD34+ cells were separated and harvested by the immunomagnetic bead method. After resuspension in EGM medium (Lonza, Cologne, Germany), the cells were seeded into the flasks and cultured at 37 °C with 5% CO_2_ in a humidity atmosphere. The culture medium was replaced 24 h to remove nonadherent cells. After culture and passage, EPCs at passage 3 were used for the further experiments [18].

### Flow cytometry analysis and immunofluorescence staining

1 × 10^6^ ASCs and EPCs at passage 3 were harvested, washed with 10% FBS/PBS, and centrifuged at 1000 rpm for 5 min to collect cell pellets.

#### Identification of ASC surface markers

FITC-labeled antibody was added to the cell suspension, incubated, centrifuged, washed, and resuspended the cells. Then, flow cytometry was used to detect the surface markers including CD73, CD90, CD45, and CD34. Moreover, the immunofluorescence staining was performed to observe the cell surface marker CD90 of ASCs. The samples were fixed and processed with 0.3% TritonX-100. After blocking with goat serum at room temperature, a fluorescent-labeled primary antibody was added, then incubated, washed, and finally counterstained with DAPI.

#### Identification of EPC surface markers

EPCs were added with FITC-labeled primary antibodies followed by incubation, centrifugation, and washing. Then, the surface markers including CD133, CD11b, CD34, and CD31 were detected by flow cytometry. In addition, EPC slides were processed with rabbit anti-rat VEGF primary antibody and incubated overnight at 4 °C. Then, the fluorescent secondary antibody was added and incubated at 37 °C for 1 h. Finally, DAPI was used for nuclear staining.

### Identification of multipluripotent differentiation capacities of ASCs and EPCs

#### Chondrogenic induction and differentiation of ASCs

When the third generation of ASCs reached a confluence rate of 80–90%, the cells were cultured in a chondrogenic differentiation medium for 3 weeks. After induction of differentiation, the cells were stained with toluidine blue, with blue as a positive result.

#### Osteogenic differentiation of ASCs

After the third-generation ASCs reached 80–90% confluence, they are cultured with an osteogenic differentiation medium for 3 weeks. Then the cells were stained with alizarin red and observed under a microscope, and the cell matrix was stained red as a positive result.

#### Adipogenic induction and differentiation of ASCs

The third-generation ASCs that reached 100% confluence were cultured in adipogenic differentiation medium A for 3 days and then replaced with adipogenic differentiation medium B. Medium A and medium B were used alternately 3 to 5 times. Finally, the sample was stained with Oil Red O and observed under a microscope. The positive result was the formation of red-stained lipid droplets in the cytoplasm of ASCs.

#### In vitro angiogenesis capability of EPCs

In a 96-well plate, per well was coated with 50 μl Matrigel, and EPC cell suspension at a concentration of 2 × 104/ml was inoculated into each well and incubated 37 °C for 12 h. Observed under an inverted microscope, the positive result is the formation of a tube-like structure.

#### Phagocytic function of EPCs

The second-generation EPCs with a confluence of 80% were processed with 4 μg/ml DilacLDL and incubated at 37 °C for 4 h. After washes with PBS and fixation with the paraformaldehyde, the sample was treated with 10 mg/l FITC-UEA-1 and incubated for 1 h, then observed under a laser confocal microscope. Dil-acLDL and FITC-UEA-1 double staining-positive cells are considered to be differentiated EPCs [19].

### Preparation of dermal extracellular matrix hydrogel

Dermal extracellular matrix (dECM) was prepared as previously described. In brief, full-thickness skin was obtained within 1 h following the slaughtering of the healthy pigs (average age 6–8 months and weight 110–120 kg) at a local slaughterhouse located in Shenyang, China. The epidermis, subcutaneous fat, and connective tissue were removed by mechanical layering to separate the dermis. The dermis was treated with the following solutions under constant agitation on an orbital shaker at 300 RPM: 0.25% trypsin at 4 °C for 6 h, three washes with deionized water for 15 min, 70% ethanol for 10 h, 3% H_2_O_2_ for 15 min, three washes with deionized water for 15 min, 1% Triton X-100 in 0.26% EDTA/0.69% Tris for 22 h with a fresh change at the 6th hour, another three washes with deionized water, 0.1% peracetic acid/4% ethanol for 2 h, two washes with PBS, and three washes with deionized water at the end.

Subsequently, dECM were frozen and lyophilized for 24 h to prepare the hydrogel. After grinding and sieving through a 40-mesh screen, the powdered ECM was then enzymatically digested in a solution of 1 mg/ml pepsin (Sigma) in 0.01 M hydrochloric acid under a constant stirring for 48 h at room temperature. Then, the acidic digest solution with a concentration of 10 mg/ml was prepared for subsequent experiments. Prior to the gelation, neutralization was performed by the addition of one tenth the digest volume of 0.1 M sodium hydroxide and one ninth the volume of 10× PBS on ice, and the neutralized solution obtained was the pre-gel. The pre-gel was placed at 37 °C for 30 min for gelation to occur.

### Live/dead staining

In the live/dead cell staining, calcein AM is capable of permeating the membrane of viable cells, where it is cleaved by intracellular esterase and produces a green fluorescence. Ethidium bromide homodimer-1 is able to enter cells with damaged membranes and bind to fragmented nucleic acids, thereby producing red fluorescence in dead cells. On the 3rd and 7th day, the cells were gently rinsed with sterile PBS and incubated with a Live/Dead Assay Kit for 20 min in an incubator. The sections were immediately transferred and imaged with an inverted fluorescence microscope with a confocal imaging system.

### CCK-8 assay to detect the proliferation activity of ASCs cultured with dECM

After being digested with trypsin, 2nd-generation ASC suspension was prepared in LG-DMEM medium containing 10% FBS, and the cell density was adjusted to 2 × 10^4^/ml. one hundred microliters of cell suspension per well was inoculated and cultured for 24 h in a 96-well culture plate. After aspirating the original medium, the cells were treated with a medium containing 1% FBS for another 24 h. Then, it was grouped according to the type of medium added, the experimental group is dECM (cells inoculated in dECM material and cultured in L-DMEM containing 10% FBS), and the control group is L-DMEM (cells inoculated and cultured in L-DMEM containing 10% FBS), with 3 repeated well per group. After culturing for 24 h, 100 μl of L-DMEM containing 10% CCK-8 solution was added to each well and incubated for 4 h at 37 °C. Afterwards, the absorbance (*A*) value at 450 nm was detected to evaluate the proliferation activity of ASCs in each group.

### Co-culture of ASCs and EPCs in vitro

To determine the optimal ratio of EPCs and ASCs in skin tissue regeneration, seven groups were divided for the next experimental observation, including ASCs alone, EPCs alone, and EPCs to ASCs at ratios of 2:1, 1: 1, 1: 2, 1: 5, and 1:10. Cells of each group were seeded in 12-well plates at a density of 1 × 10^5^ cells per well and induced with the dECM medium (cells inoculated in dECM material and cultured in L-DMEM containing 10% FBS) or L-DMEM (cells inoculated and cultured in L-DMEM containing 10% FBS) for 7 days, which was prepared detecting the expression of CK19 and vimentin by immunofluorescence.

### Matrigel tubule formation assay

According to cell composition, this assay was divided into 3 groups, namely ASC, EPC, ASC+EPC groups. Prior to the assay, the Matrigel (BD Corporation, USA) was moved from − 20 to 4 °C to fully melt. Then, 50 μl of Matrigel was added to each well of a 96-well plate and placed at 37 °C for 2 h to coagulate. The cells of the three groups were seeded to the 96-well plate containing matrigel (5 duplicate wells per group), placed in a 37 °C, 5% CO_2_ incubator, and observed under a light microscope every 3 h. When obvious blood vessels were formed, photographs were taken immediately to record. The number and relative length of each component tube were analyzed by the Image-Pro Plus 6.0 software.

### Quantitative reverse transcription-polymerase chain reaction

The cell grouping in qRT-PCR is the same as the tubule formation experiment. At first, the three groups of cells were respectively seeded and cultured on a dECM induction medium for 7 days. After that, total RNA was extracted from the cells of the different groups using TRIzol Reagent (Invitrogen) according to the manufacturer’s instructions. Reverse transcription of total RNA into cDNA was performed by RT-PCR (Invitrogen) using the reverse transcription first chain synthesis system. PCR amplification with specific primers was employed to analyze the expression of epidermal cell marker genes including keratin (CK5, CK19), Col I, Col III, vascular endothelial growth factor (VEGF), and von Willebrand factor (vWF). Following the manufacturer’s instructions, real-time PCR was performed using the SYBR GREEN PCR Master Mix on ABI PRISM 7500 sequence detection system. PCR conditions were 94 °C 1 min, 95 °C 30 s, 58 °C 40 s, and 72 °C 1 min 30 s with a total of 40 cycles. At last, all reactions were repeated three times and normalized to GAPDH. Comparative CT was calculated to evaluate the relative difference of PCR results of each group.

### Establishment of wound model

Forty-eight male Sprague-Dawley rats (8 weeks old) weighing 200–250 g were anesthetized by intraperitoneal injection of 1% sodium pentobarbital solution (40 mg/kg body weight). Rats were randomly divided into four groups (*n* = 10 each group) according to the construct used to repair the wound: Black, ECM+EPCs (ECM+E), ECM+ASCs (ECM+A), and ECM+ASCs+EPCs (ECM+A+E). The cells of each group were mixed with sterilized dECM hydrogel at a concentration of 1 × 10^6^/ml to prepare an injectable gel scaffold for use. Each wound was covered with a mixed gel material of 2 ml hydrogel and cells, and bandaged after surgery. The rats were given an intramuscular injection of anti-inflammatory and analgesic drugs and were kept separately for observation to avoid biting each other. To evaluate the therapeutic effect, the wound closure of rats was observed and recorded. At 7 and 14 days after the operation, 6 rats were taken from each group. After anesthesia, the wound was covered with a sterile plastic film and the size was drawn with a pencil. The drawn film was scanned into a computer, and the Image-Pro Plus 6.0 image analysis software was used to analyze the contour size and calculate the wound closure rate according to the following formula: (original wound area − wound area after treatment)/original wound area × 100%.

### Histological analysis

On the 14th day after operation, the rats in four groups were anesthetized and sacrificed. The middle part of the repair area in each group was trimmed for histological analysis. Following fixation in 4% paraformaldehyde for 24 h, dehydrated in a graded series of ethanol, embedded in paraffin, and cut into 4 μm sections. After deparaffinization, hematoxylin and eosin (HE) staining was performed. A near-surface field of view was randomly selected, and a low-magnification field (10 × 10) was used to choose the area with a large number of inflammatory cells (neutrophils, eosinophils, basophils, lymphocytes, and monocytes). Then 5 fields in the high-magnification field (10 × 40) were selected to observe and count the number of inflammatory cells in the field, and the average value was calculated. In addition, Masson trichrome staining was used to analyze the morphological characteristics of collagen fibers and the healing of skin wounds.

### Immunofluorescence analysis of vascularization

Tissue sections on the 14th day after surgery were deparaffinized with xylene, dehydrated by ethanol, antigen retrieval, and serum blocking, and the sections were added with rabbit anti-CD31 polyclonal antibody (Abcam) to label vascular endothelial cells. Three areas with high microvessel density marked by CD31 were randomly selected in the low magnification field of view (10 × 10), and then the microvessel density in 5 fields in each area was counted in the high magnification field of view (10 × 20), and the average value was calculated.

### Statistical analysis

The test results were expressed as mean ± standard deviation, and statistical analysis was performed by the SPSS 21.0 software (IBM, Armonk, New York, USA). *T* test was used for the comparison of the data of the two groups, and one-way ANOVA was for the samples of multiple groups. *p* < 0.05 was considered as statistically significant.

## Results

### Detection and characterization of ASCs and EPCs

**Fig. 1:**
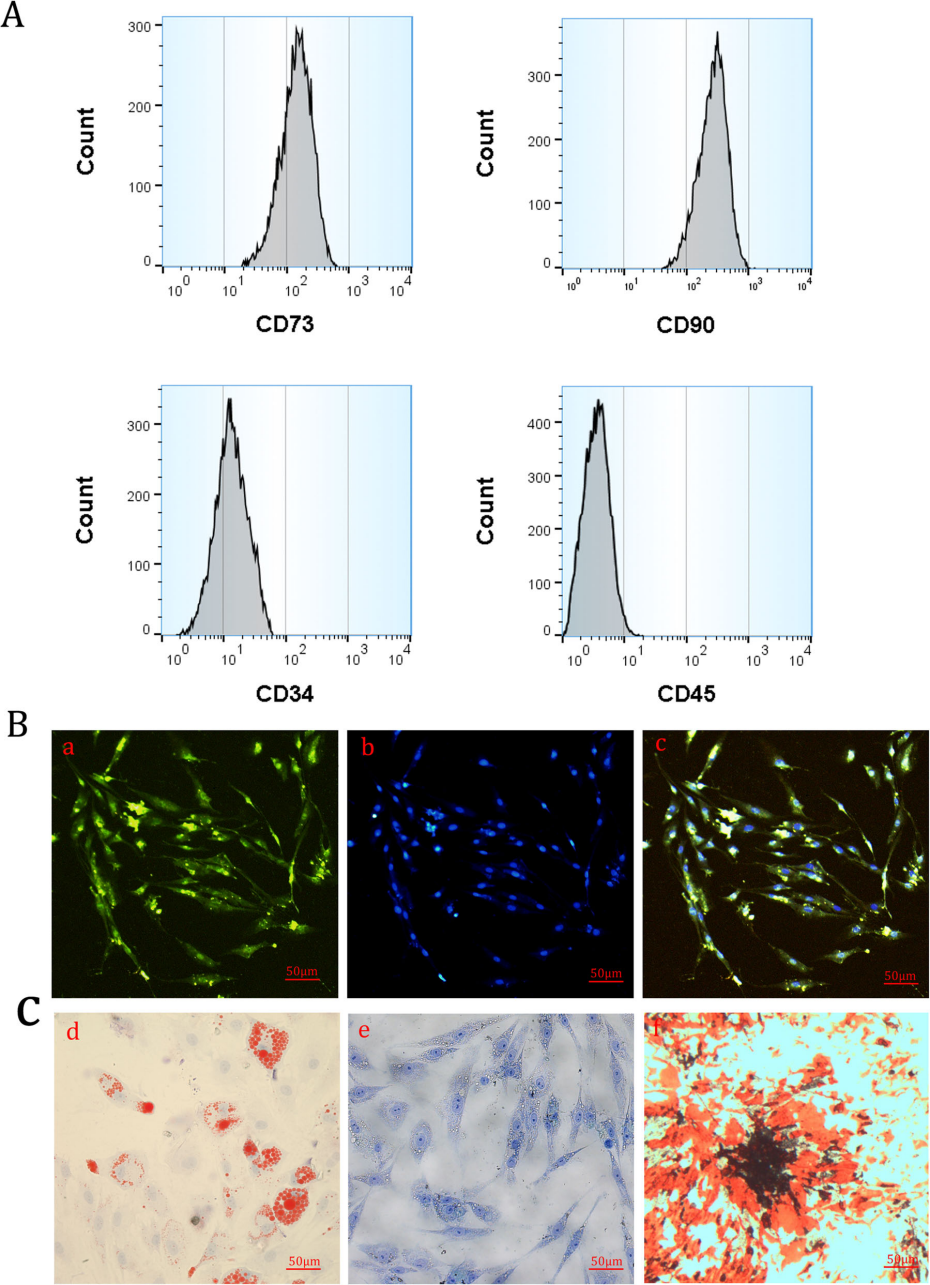
Detection and characterization of ASCs. **A** Surface markers of ASCs in flow cytometry. **B** ASC immunofluorescence staining: (a) CD90, (b) DAPI, and (c) CD90 + DAPI. **C** Identification of the multipluripotent differentiation capacities of ASCs: (d) adipogenic differentiation, (e) chondrogenic differentiation, and (f) osteogenic differentiation

The flow cytometry of ASCs demonstrated that ASCs are positive for CD73 and CD90 and negative for CD45 and CD34 (Fig. 1a). The immunofluorescence staining also exhibited that CD90 was positive in ASCs (Fig. 1b). After induced differentiation, ASCs have osteogenic, adipogenic, and chondrogenic differentiation capabilities (Fig. 1c).

**Fig. 2:**
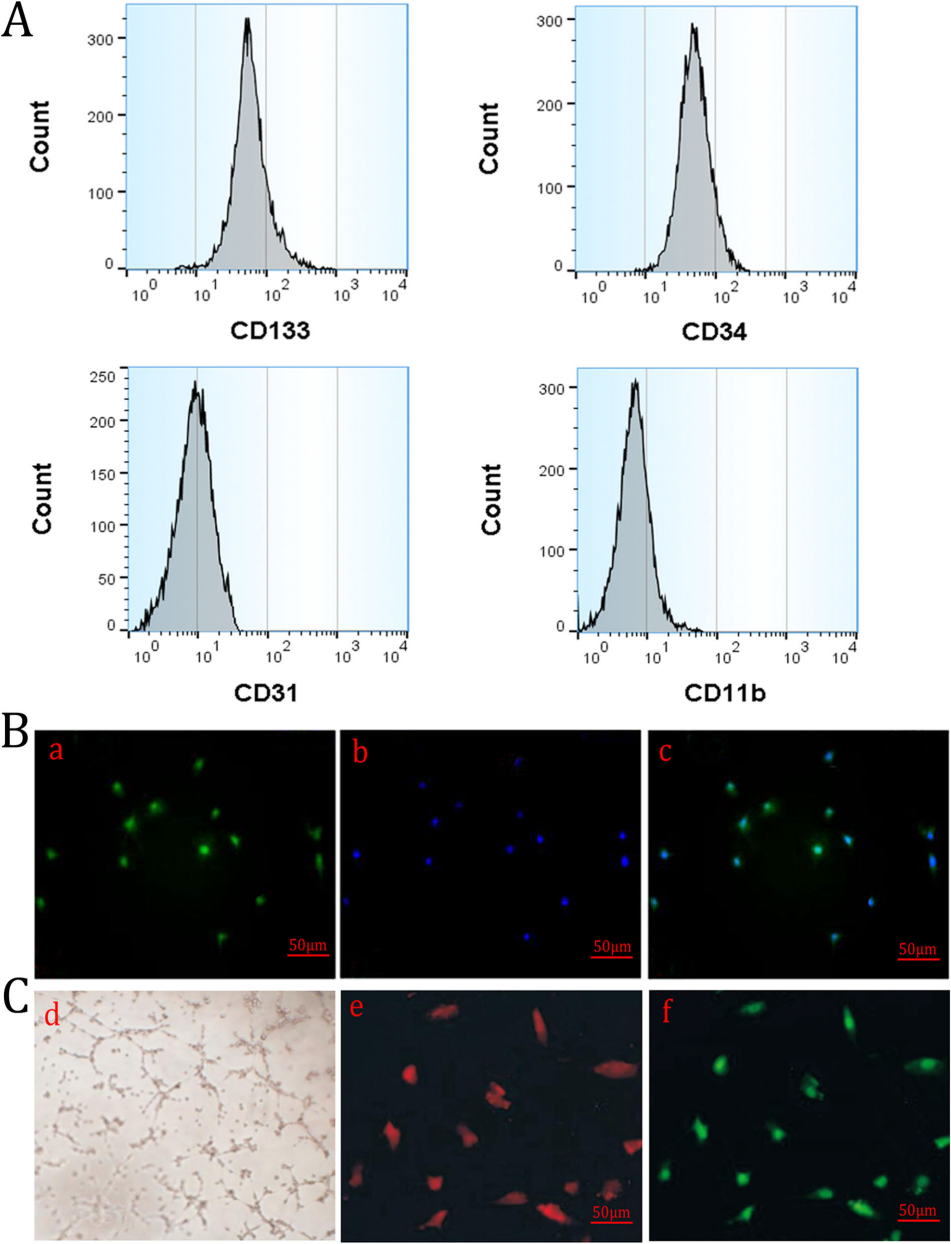
Detection and characterization of EPCs. **A** Surface markers of EPCs in flow cytometry. Low expression of CD31 and CD11b and high expression of CD133 and CD34. **B** EPC immunofluorescence staining: (a) VEGF, (b) DAPI, and (c) VEGF + DAPI. **C** Identification of the multipluripotent differentiation capacities of EPCs: (d) Matrigel tubule formation assay, (e) Dil-ac-LDL, and (f) lectin

EPCs are positive for CD133 and CD34 and negative for CD31 and CD11b in flow cytometry (Fig. 2a). Meanwhile, it was shown that VEGE immunofluorescence staining was positive (Fig. 2b). Moreover, the cells seeded in a 96-well plate coated with Matrigel could form a “lumen-like” structure, and take up Dil-ac-LDL and lectin (Fig. 2c), indicating that the target cells are EPCs.

### dECM hydrogel promotes ASC proliferation and has thermosensitive properties

**Fig. 3:**
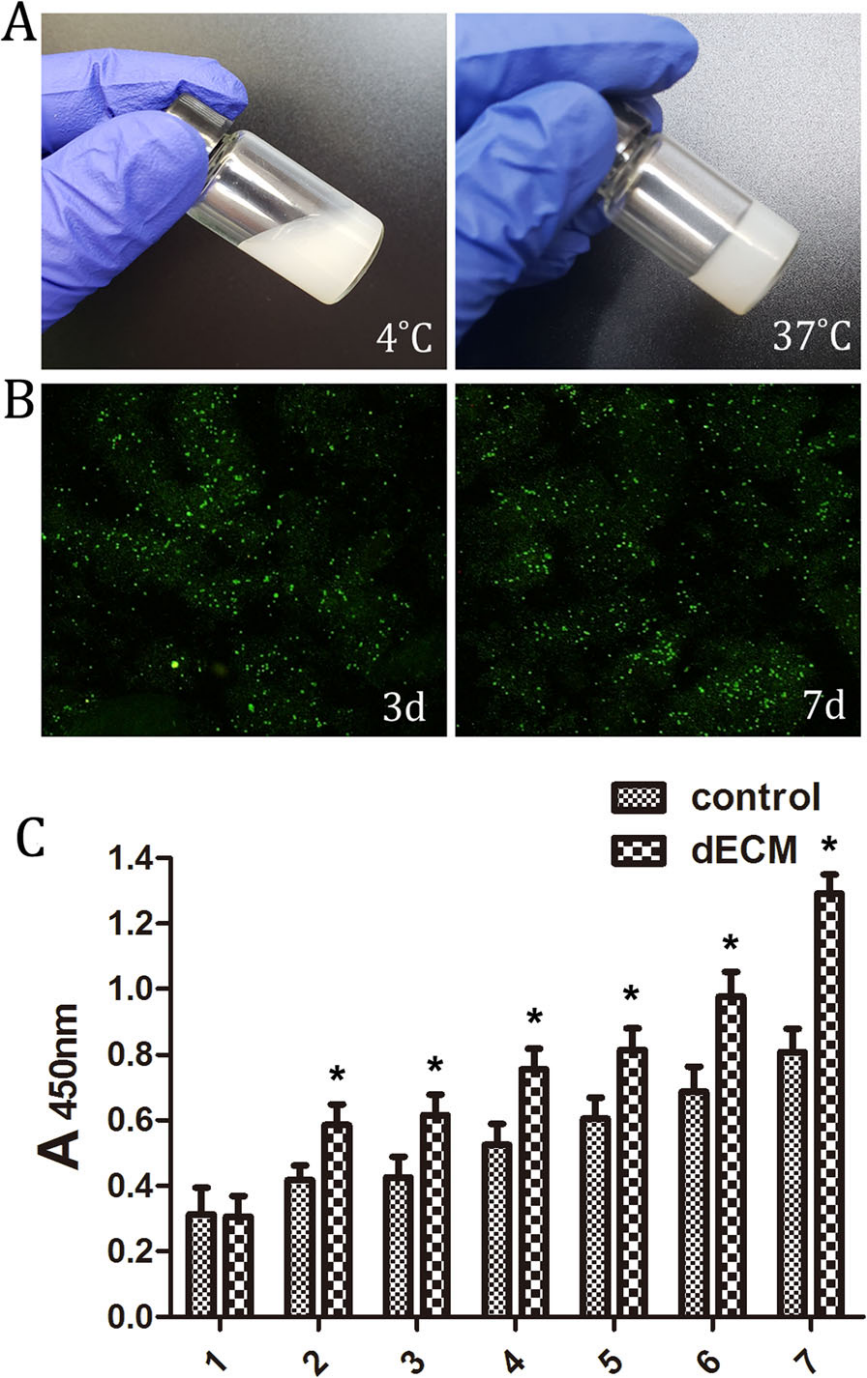
Evaluation and characterization of dECM hydrogel. **a** dECM in a liquid state at 4 °C and in a gel state at 37 °C. **b** ASCs were inoculated and cultured with dECM as a scaffold, and cell viability was detected with live/dead staining on the 3rd and 7th day. **c** CCK-8 assay was used to detect the proliferative capability of ASCs. The proliferation of ASCs in the dECM group was significantly higher than that in the control group from the 2nd to 7th day (Fig. 3c), *p < 0.05

Stem cells were inoculated and cultured with dECM as a scaffold, and cell viability was detected at two time points, 3and 7 days. The results shown in Fig. 3b demonstrates that the cell viability was good, indicating that dECM has no cytotoxicity. Meanwhile, it was found that dECM has thermosensitive properties. The dECM pre-gel is liquid at low temperatures and transformed into a gel state at 37 °C (Fig. 3a), which made it an injectable scaffold material for skin defect repair.

CCK-8 assay was used to detect the proliferative capability of ASCs. The proliferation of ASCs in the dECM group was significantly higher than that in the control group from the 2nd to the 7th day (Fig. 3c) (*p* < 0.05), indicating that dECM hydrogel could promote the proliferation of ASCs.

### Determination of the optimal ratio of ASC/EPC co-culture in vitro

To determine the optimal ratio of ASCs/EPCs in vitro co-culture, the expression of epidermal cell markers CK19 and vimentin in different ratios of the ASC/EPC co-culture system was compared after culture in the dECM medium and L-DMEM (Table 1). The comparison results showed that the expression of CK19 and vimentin in ASCs/EPCs co-cultured at a cell ratio of 1:1 in a dECM medium were significantly higher than that of other groups. With the same cell ratio, the expression of CK19 and vimentin in the dECM medium was higher than that of the L-DMEM group.

**Table 1:** The detection of CK19 and vimentin expression by immunofluorescence staining

		ASCs	EPCs	2:1	1:1	1:2	1:5	1:10
CK19	ECM	6.16 ± 0.23*	5.32 ± 0.32*	11.73 ± 0.44*	17.24 ± 0.35*^b^	10.51 ± 0.38*	7.33 ± 0.42*	6.67 ± 0.56*
	CM	2.55 ± 0.45	2.05 ± 0.31	4.23 ± 0.41	6.33 ± 0.52	6.14 ± 0.42	5.37 ± 0.38	3.66 ± 0.42
Vimentin	ECM	3.36 ± 0.41*	2.68 ± 0.32*	6.42 ± 0.31*	11.38 ± 0.21*^b^	5.82 ± 0.37*	4.74 ± 0.26*	3.62 ± 0.25*
	CM	1.45 ± 0.45	1.17 ± 0.27	2.63 ± 0.24	5.71 ± 0.36	3.51 ± 0.32	3.05 ± 0.31	2.51 ± 0.23

*p < 0.05 vs. the CM group; ^b^*P* < 0.05 vs. the other cell proportion groups (2:1, 1:1, 1:2, 1:5, and 1:10 represent the different cell proportions of EPCs: ASCs)

### ASC/EPC co-culture in vitro promotes the expression of dermal tissue markers and angiogenesis-related genes

**Fig. 4:**
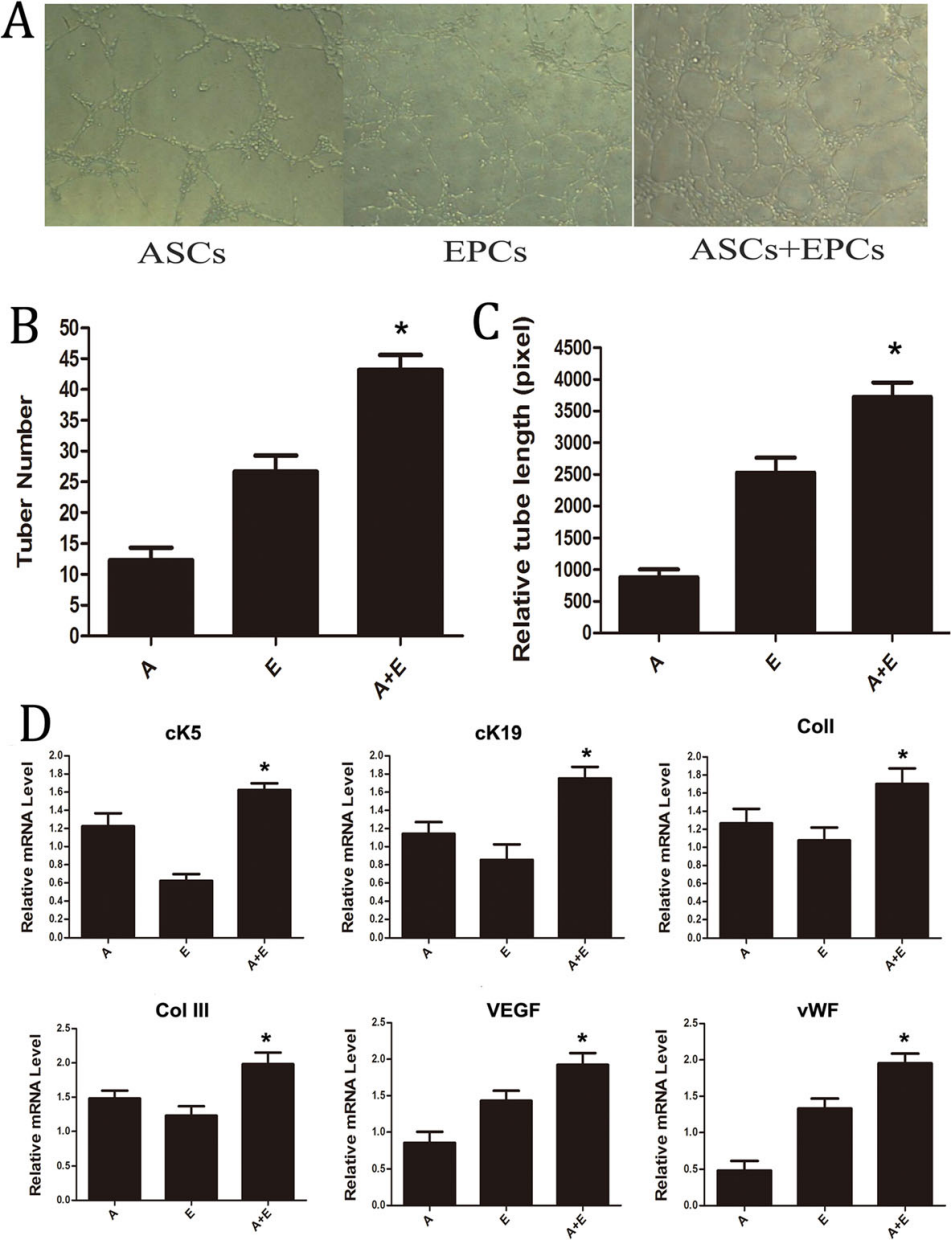
Detection and analysis of ASC and EPC co-culture in vitro. **a** The results of the Matrigel tubule formation assay under a light microscope are presented. **b**:Comparison of the amount of tubular structures among the three groups, *p < 0.05. **c** Comparison of the relative length of tubular structure among the three groups, *p < 0.05. **d** RT-PCR detection of mRNA expression of skin-related genes (Ck5, Ck19, ColI, and Col III) and angiogenesis-related genes (VEGF and vWF). (A: ASCs, E: EPCs), *p < 0.05, A+E group compared with all other groups

Matrigel tubule formation assay demonstrated that the density of tube structure in the ASC/EPC co-culture group was dramatically higher than that in the ASC alone or EPC alone group (Fig. 4a). Moreover, the amount of tubular structures increased significantly in the ASC/EPC co-culture group compared with the single-cell groups (*p* < 0.05) (Fig. 4b). And the length of the tubular structure formed in the co-culture group was also significantly longer than that of the other two groups (*p* < 0.05) (Fig. 4c).

In order to further study whether the co-culture of ASCs and EPCs affects the expression of epidermal cell marker and endothelial cell marker genes, RT-PCR was used to analyze the main components of dermal tissues including epidermal cell and collagen corresponding markers Ck5, Ck19, Col I, and Col III and the expression of endothelial cell marker genes, such as VEGF and vWF. The results showed that the expression levels of Ck5, Ck19, Col I, and Col III in the ASC/EPC group (A+E group) were significantly higher than those in the ASC group (A group) and EPC group (E group) (Fig. 4d), suggesting that the co-culture of ASCs/EPCs could increase the expression of epidermal cell and collagen marker genes. In addition, the mRNA levels of endothelial cell marker genes VEGF and vWF were dramatically higher in the A+E group than in the A group and E group (Fig. 4d), which indicated that the co-culture of ASCs/EPCs could increase the expression of angiogenesis-related genes. The above results demonstrated that the co-culture of ASCs/EPCs could enhance the expression of dermal tissue markers and vascular endothelial cell genes.

### Co-culture of ASCs and EPCs enhances the repair of full-thickness skin defects in rats

**Fig. 5:**
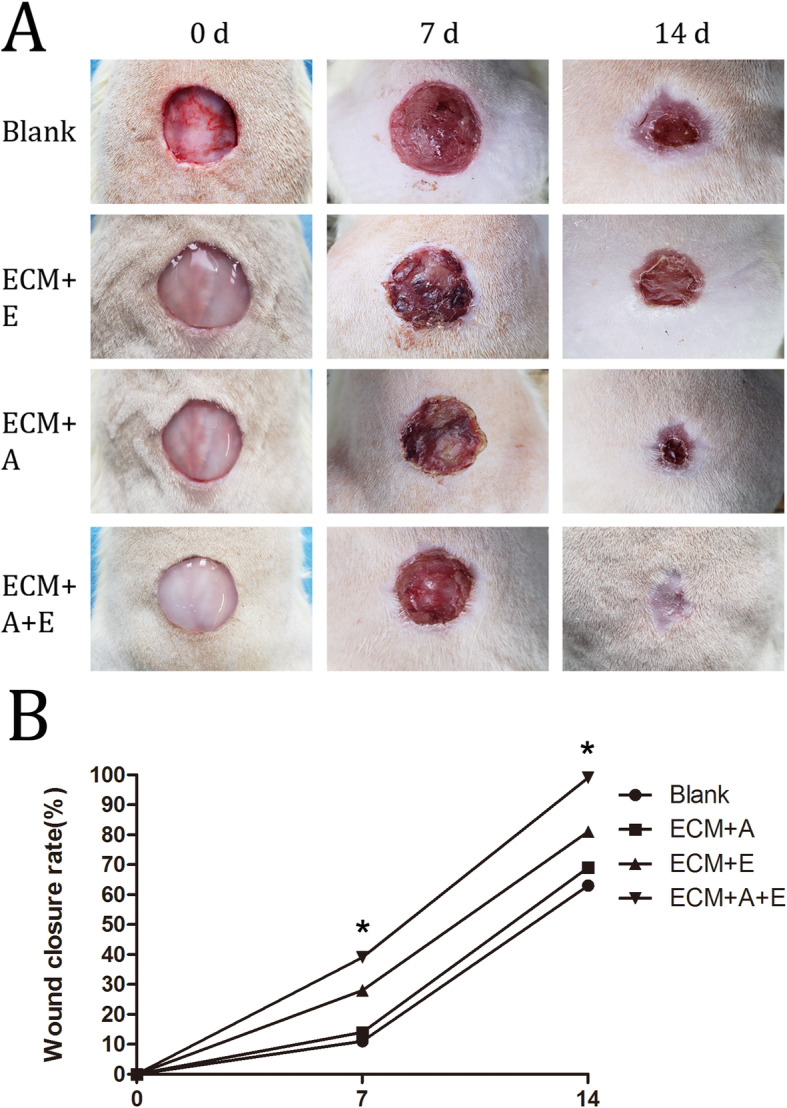
Co-culture of ASCs and EPCs enhances the repair of full-thickness skin defects in rats. **a** Comparison of wound closure in each group at days 0, 7, and 14 after the operation. **b** Quantitative evaluation of wound closure rate on days 0, 7, and 14 post-treatment. *p < 0.05, the ECM+A+E group compared to all other groups

In the comparison of wound healing at different time points after the operation, it was observed that the wound healing rate was faster in the ECM+A+E group. (Fig. 5a). Seven days after treatment, the wound healing rate of the ECM+A+E group (48.12% ± 2.35%) was significantly higher than that of the other groups (blank group, 12.32% ± 2.52%; ECM+E group, 15.67% ± 4.25%; and ECM+A group, 26.05% ± 2.43%; all *p* < 0.05). Fourteen days after treatment, the wound in the ECM+A+E group was almost closed, and the healing rate (98.26% ± 1.41%) was also remarkably higher than other groups (blank group, 62.16% ± 1.28%; ECM+E group, 68.67% ± 2.31%; and ECM+A group, 78.21% ± 3.15%; all *p* < 0.05) (Fig. 5b). In terms of healing rate, the ECM+A group is higher than the blank group and ECM+E group, but lower than the ECM+A+E group. In addition, compared with other groups, the newly formed skin color and shape of the ECM+A+E group are remarkably closer to normal skin. On the contrary, other groups, especially the blank group and ECM+E group, had obvious scar tissue formation and contraction.

**Fig. 6:**
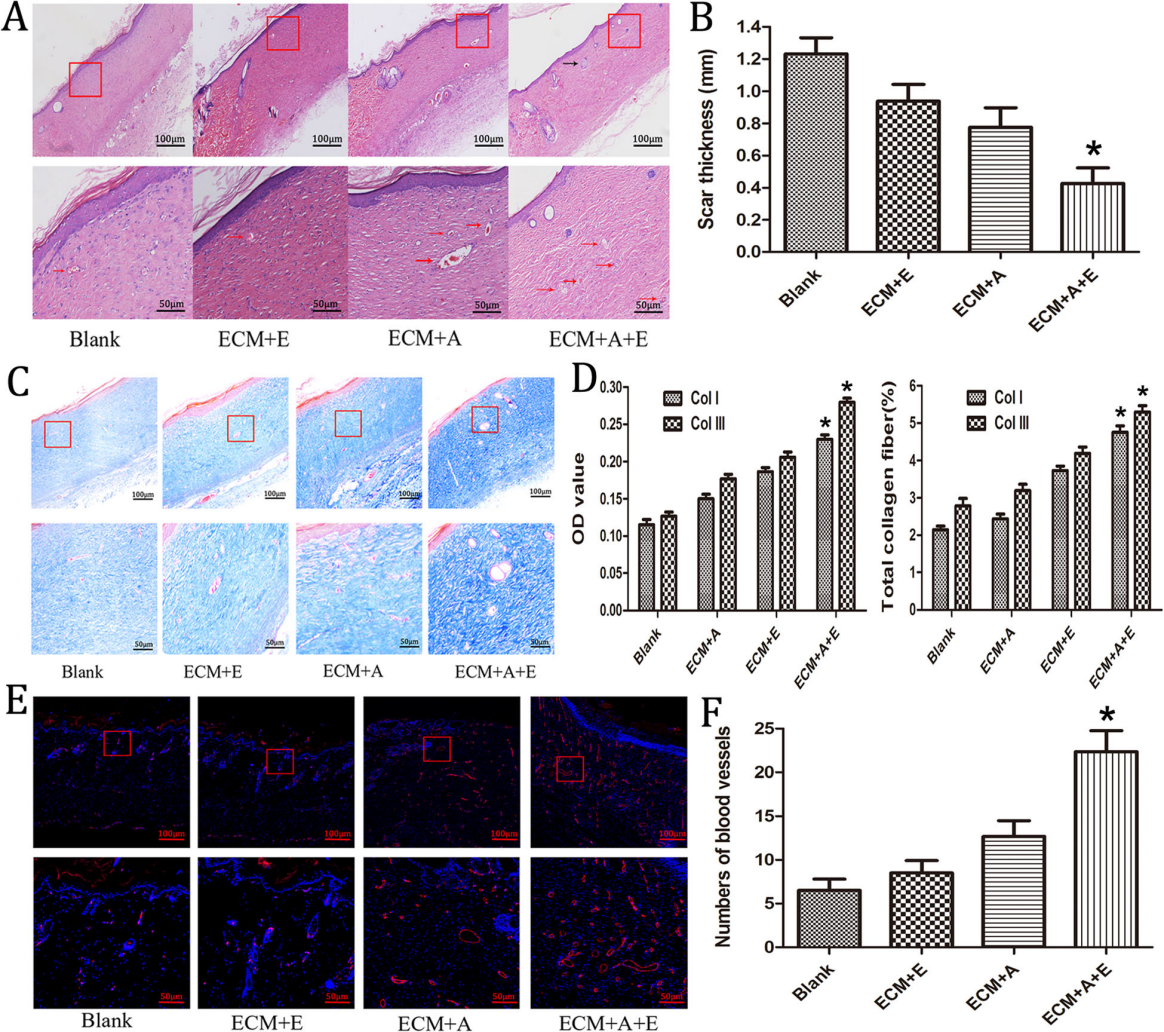
Microscopic observation of postoperative skin regeneration. **a** Analysis of HE staining of the wound on the 14th day after the operation. The black arrow indicates the skin appendages formed in the ECM+A+E group. The red arrows show the newly formed blood vessels in each group, and the number of new blood vessels in the ECM+A+E group is significantly more than that in the other groups. **b** Quantitative analysis of regenerated skin scar thickness, *p < 005, ECM+A+E group compared with all other groups. **c** Masson staining of regenerated skin 14 days after surgery. **d** OD value of Col I and Col III in each group. Total amount of collagen fibers in each group.*p < .005, the ECM+A+E group compared to all other groups. **e** CD31 immunofluorescence detection of regenerated skin tissue. The blood vessels are shown by red fluorescence. The number of new blood vessels in the ECM+A+E group is significantly more than that of the other groups. **f**: Quantitative analysis of the number of regenerated blood vessels, *p < 005, compare ECM+A+E group with other groups

The HE staining results of the regenerated skin tissue showed that the thickness of the newly formed epithelium in the ECM+A+E group was greater than that of the other groups, and there were skin appendages formed as shown in Fig. 6a. Meanwhile, it was found that there were different degrees of angiogenesis in each group, and the ECM+A+E group was significantly more than the other groups (Fig. 6a). In terms of the thickness of scar tissue in regenerated skin, the ECM+A+E group was significantly smaller than the other groups as shown in Fig. 6b (*p* < 0.05).

The regeneration of collagen tissue was evaluated by Masson staining, which indicated that the ECM+A+E group was significantly more than other groups as shown in Fig. 6c. The OD values of Col I and Col III in each group were quantitatively measured, suggesting that the average collagen content of the ECM+A+E group was significantly higher than that of the other groups as shown in Fig. 6d (*p* < 0.05). The total content of Col I and Col III was measured for comparison and analysis, which demonstrated that the result in the ECM +A+E group was also dramatically higher than that of the other groups as shown in Fig. 6d (*p* < 0.05).

CD31 immunofluorescence assay was performed on the regenerated skin tissue, which showed that the blood vessel fluorescence staining range in the ECM+A+E group was larger than that in the other groups as shown in Fig. 6e (the red color in the figure is the blood vessel morphological staining). According to the quantitative analysis shown in Fig. 6f, the number of newly formed blood vessels in the ECM+A+E group was significantly higher than that in the other groups (*p* < 0.05).

## Discussion

There are still some problems to be solved in the reconstruction of large-scale full-thickness skin defects, including slow wound healing, obvious scars, and obvious skin tissue shrinkage, which is an attribute to the lack of vascularized skin tissue regeneration, affecting the repair effect of skin defects [20]. To construct skin tissues accompanying the vascular system, our team co-cultured ASCs and EPCs to establish a dual stem cell system. This study found that the ASC/EPC co-culture system could enhance the expression of skin and angiogenesis genes in vitro, and by supporting the regeneration of vascularized skin, it significantly accelerated the wound healing of skin defects in vivo.

EPCs are the precursor cells of vascular endothelial cells, which have the ability to proliferate, migrate, and differentiate into cells arranged along the vascular cavity, and can be isolated from the peripheral blood and spleen. ASCs also have high proliferation and growth characteristics and multi-differentiation potential, which can be extracted from autologous subcutaneous fat and are the most easily obtained autologous stem cells. But so far, specific markers for each cell type are still lacking. Many surface proteins have been used to identify rat ASCs, including CD73, CD90, CD105, and CD44 [21, 22]. In this study, CD73 and CD90 are used as positive markers for ASCs. The results showed that ASCs expressed CD73- and CD90-positive cell surface proteins, and CD45- and CD34-negative cell surface proteins. In EPCs, CD34 and CD133 were detected, which were highly expressed in EPCs, but not expressed after EPCs differentiated into mature vascular endothelial cells [23]. In the meantime, CD31, which is not expressed in EPCs but highly expressed in mature endothelial cells, and CD11b, which is expressed in monocytes but not in EPCs, were also detected [24–27]. It was shown that EPCs expressed CD133 and CD34 with positive surface protein profiles, and CD11b and CD31 had negative surface protein profiles. These results confirmed the phenotype of ASCs and EPC, suggesting that the abovementioned markers could be used for the identification of ASCs and EPCs.

In this study, a porcine acellular dermal ECM was prepared according to the protocol proposed by Matthew T. Wolf, as an injectable thermosensitive gel scaffold material for repairing skin defects. It was found that the dermal ECM can be used as a stem cell induction medium without cytotoxicity, in which cells could survive continuously for more than 7 days and exhibit proliferation. To further determine the optimal ratio of ASC/EPC co-culture, 7 groups of different ratios of cell co-culture experiments in vitro were designed and grouped according to the ratio of EPCs in cells from less to more. The purpose is to study the effect of different EPCs ratios on wound healing to determine the best co-culture ratio. It was found that when the ratio of ASCs/EPCs was 1:1, the expression of CK19 and vimentin was significantly higher than that of the other groups. Moreover, the expression of CK19 and vimentin in the ECM medium group was also dramatically higher than that of the L-DMEM group, indicating that the co-culture of ASCs/EPCs induced by ECM at a ratio of 1:1 is more conducive to the differentiation of stem cells into epidermal-like cells. After determining the optimal ratio, RT-PCR analyzed the expression of ASCs skin marker genes CK5, CK19, Col I, Col III, and endothelial marker genes VEGF and vWF, which showed that the ASC/EPC co-culture system could increase the expression of skin-related and angiogenesis-related genes. In addition, it was found in Matrigel tubule formation assay that ASC/EPC co-culture could improve the angiogenesis ability of cell lines.

On the basis of in vitro experiments, in vivo experiments on repairing rat skin defects were further carried out. The animal experiment was designed into 4 groups: blank control group, ECM+E group, ECM+A group, and ECM+A+E group. The healing area was measured and analyzed at 7 and 14 days after the operation. The results clearly showed that the repair speed of the skin defect area in the ECM+A+E group was significantly faster than the other groups. In terms of healing rate, the ECM+A group was higher than the blank group and the ECM+E group, but lower than the ECM+A+E group. In addition, the color and shape of the newly formed skin in the ECM+A+E group were significantly closer to normal skin, while there were obvious scar tissue formation and contraction in other groups, especially the blank group and ECM+E group. HE staining showed that the angiogenesis density in the ECM+A+E group was higher than that in the other three groups, and a small amount of skin appendage formation was also detected, which was rarely seen in other groups. The thickness of the new skin tissue in the ECM+A+E group was greater than that of the other groups, and the scar thickness was also significantly less than that of the other groups. Masson staining confirmed that the production of Col I and Col III in the ECM+A+E group was greater than that in the other groups. Furthermore, it was confirmed in CD31 immunofluorescence analysis that the number and length of new blood vessels in the defect area of the ECM+A+E group were greater than those of the other three groups. These results demonstrated that ECM+A+E group achieved more effective vascularized skin regeneration. The possible reason for this phenomenon is that the vascularization of the regenerated skin in the ECM+A group and ECM+E group is relatively small, and it only depends on the ingrowth of host blood vessels. However, the distance between the blood vessels of the host tissue and the center of the skin defect is far and insufficient to achieve skin regeneration, especially for large-scale skin defects. Therefore, nutrients, metabolites, and other molecules are unable to be transported to the central area of the defect, which severely hinders skin regeneration [28]. In the ASCs/EPCs group, EPCs directly increased vascular invasion and promoted the differentiation of ASCs into the dermal direction. ASCs could promote the recruitment of EPCs and enhance the ability of EPCs to form blood vessels. This interaction accelerated the formation of vascularized skin, making nutrients, cytokines, and other molecular factors involved in the skin healing process easier to access to the wound site. The results also showed that the implanted EPC/ASC co-culture system had a synergistic effect in comparison with the individual ASCs. The co-culture group significantly promoted skin tissue formation, which may be attributed to increased vascularization, leading to better recruitment of skin progenitor cells and other cytokines involved in skin healing. Vascularization is considered a necessary condition for skin formation, and insufficient vascularization leads to impaired skin formation or delayed skin healing [29]. Previous studies have shown that ASCs could produce soluble cytokines such as vascular endothelial growth factor (VEGF) and promote endothelial cell migration through autocrine or paracrine action, which contributes to cell rearrangement and tubular network formation [30]. VEGF is recognized as the main promoter of endothelial cell migration and tubular network formation [31]. The activation of VEGF can induce phosphorylation, participate in signal transduction, promote endothelial cell migration, and promote angiogenesis. In this process, the PI3K-Akt and MAPK pathways are the most important signal pathways for cytokine-cytokine receptor interaction, which are essential for damage repair [32–34]. Moreover, it was detected that the expression of VEGF and its receptors in the ASC/EPC co-culture group was upregulated. This may be due to the production of VEGF and other cytokines and the activation of the corresponding signal pathways by ASCs in the co-culture group, which facilitated the migration of vascular endothelial cells and the formation of tubular structures by through autocrine and paracrine, thereby increasing the formation of blood vessels. However, the molecular mechanism of this synergistic signaling pathway in the formation of vascularized skin in the ASC/EPC co-culture system is still unclear, which is also the focus of the next step of our study group.

## Conclusion

In summary, this study proved that the ASC/EPC co-culture cell system could synergistically promote the formation of vascularized skin, thereby realizing the repair of large-scale skin defects, which is unable to be achieved by other single seed cells. In addition, the co-culture cell system has the advantages of easy availability and low cost compared with stem cell angiogenesis-related gene transfection and other pro-vascularization methods, so it has a good application perspective. Based on the above results, we believe that it is feasible to use ASC/EPC co-culture system to replace traditional single-seed cells as a potential method of vascularized skin regeneration.

## Data Availability

The datasets used and/or analyzed during the current study are available from the corresponding author on reasonable request.

## Abbreviations

EPCs: Endothelial progenitor cells
ASCs: Adipose-derived mesenchymal stem cells
dECM: Dermal extracellular matrix
CK5: Cytokeratin 5
CK19: Cytokeratin 19
Col I: Type I collagen fibers
Col III: Type III collagen fibers
VEGF: Vascular endothelial growth factor
vWF: von Willebrand factor

## References

[1] Steffens D (2017). Treatment of a burn animal model with functionalized tridimensional electrospun biomaterials. J Biomater Appl.

[2] Huging M (2017). The effect of wound dressings on a bio-engineered human dermo-epidermal skin substitute in a rat model. J Burn Care Res.

[3] Motamed S (2017). Cell-based skin substitutes accelerate regeneration of extensive burn wounds in rats. Am J Surg.

[4] Caliari-Oliveira C (2016). Xenogeneic mesenchymal stromal cells improve wound healing and modulate the immune response in an extensive burn model. Cell Transplant.

[5] Weng T (2020). Regeneration of skin appendages and nerves: current status and further challenges. J Transl Med.

[6] An Y (2018). Autophagy promotes MSC-mediated vascularization in cutaneous wound healing via regulation of VEGF secretion. Cell Death Dis.

[7] Zhou ZQ (2019). Adipose extracellular matrix promotes skin wound healing by inducing the differentiation of adipose-derived stem cells into fibroblasts. Int J Mol Med.

[8] Luo Y (2019). Autograft microskin combined with adipose-derived stem cell enhances wound healing in a full-thickness skin defect mouse model. Stem Cell Res Ther.

[9] Carmeliet P (2005). Angiogenesis in life, disease and medicine. Nature.

[10] Lin Y (2000). Origins of circulating endothelial cells and endothelial outgrowth from blood. J Clin Invest.

[11] Urbich C, Dimmeler S (2004). Endothelial progenitor cells: characterization and role in vascular biology. Circ Res.

[12] Caiado F, Dias S (2012). Endothelial progenitor cells and integrins: adhesive needs. Fibrogenesis Tissue Repair.

[13] Suh W (2005). Transplantation of endothelial progenitor cells accelerates dermal wound healing with increased recruitment of monocytes/macrophages and neovascularization. Stem Cells.

[14] Li P, Guo X (2018). A review: therapeutic potential of adipose-derived stem cells in cutaneous wound healing and regeneration. Stem Cell Res Ther.

[15] Lee DE, Ayoub N, Agrawal DK (2016). Mesenchymal stem cells and cutaneous wound healing: novel methods to increase cell delivery and therapeutic efficacy. Stem Cell Res Ther.

[16] Steiner D (2017). Cocultivation of mesenchymal stem cells and endothelial progenitor cells reveals antiapoptotic and proangiogenic effects. Cells Tissues Organs.

[17] Gangadaran P (2017). Extracellular vesicles from mesenchymal stem cells activates VEGF receptors and accelerates recovery of hindlimb ischemia. J Control Release.

[18] Jia Y (2019). Exosomes secreted by endothelial progenitor cells accelerate bone regeneration during distraction osteogenesis by stimulating angiogenesis. Stem Cell Res Ther.

[19] Asahara T (1997). Isolation of putative progenitor endothelial cells for angiogenesis. Science.

[20] Demidova-Rice TN, Hamblin MR, Herman IM (2012). Acute and impaired wound healing: pathophysiology and current methods for drug delivery, part 1: normal and chronic wounds: biology, causes, and approaches to care. Adv Skin Wound Care.

[21] Quarles LD (2012). Skeletal secretion of FGF-23 regulates phosphate and vitamin D metabolism. Nat Rev Endocrinol.

[22] Granero-Molto F (2008). Role of mesenchymal stem cells in regenerative medicine: application to bone and cartilage repair. Expert Opin Biol Ther.

[23] Yin AH (1997). AC133, a novel marker for human hematopoietic stem and progenitor cells. Blood.

[24] Stein A (2008). Circulating endothelial progenitor cells decrease in patients after endarterectomy. J Vasc Surg.

[25] Salven P (2003). VEGFR-3 and CD133 identify a population of CD34+ lymphatic/vascular endothelial precursor cells. Blood.

[26] Walter DH, Dimmeler S (2002). Endothelial progenitor cells: regulation and contribution to adult neovascularization. Herz.

[27] McCarty RC (2009). Characterisation and developmental potential of ovine bone marrow derived mesenchymal stem cells. J Cell Physiol.

[28] Reichenberger MA (2012). Adipose derived stem cells protect skin flaps against ischemia-reperfusion injury. Stem Cell Rev Rep.

[29] Singer AJ, Clark RA (1999). Cutaneous wound healing. N Engl J Med.

[30] Wang X (2018). Exosomes from adipose-derived stem cells promotes VEGF-C-dependent lymphangiogenesis by regulating miRNA-132/TGF-β pathway. Cell Physiol Biochem.

[31] Alexander RA (2012). VEGF-induced endothelial cell migration requires urokinase receptor (uPAR)-dependent integrin redistribution. Cardiovasc Res.

[32] Sun L, et al. The CLC-2 chloride channel modulates ECM synthesis, differentiation, and migration of human conjunctival fibroblasts via the PI3K/Akt signaling pathway. Int J Mol Sci. 2016;17(6):910. 10.3390/ijms17060910 PMC4926444 27294913

[33] Ohki Y (2005). Granulocyte colony-stimulating factor promotes neovascularization by releasing vascular endothelial growth factor from neutrophils. FASEB J.

[34] Shibuya M, Claesson-Welsh L (2006). Signal transduction by VEGF receptors in regulation of angiogenesis and lymphangiogenesis. Exp Cell Res.

